# Distribution of Pandemic Influenza Vaccine and Reporting of Doses Administered, New York, New York, USA

**DOI:** 10.3201/eid2004.131114

**Published:** 2014-04

**Authors:** Roopa Kalyanaraman Marcello, Vikki Papadouka, Mark Misener, Edward Wake, Rebecca Mandell, Jane R. Zucker

**Affiliations:** New York City Department of Health and Mental Hygiene, Queens, New York, USA (R.K. Marcello, V. Papadouka, M. Misener, E. Wake, R. Mandell, J.R. Zucker);; Centers for Disease Control and Prevention, Atlanta, Georgia, USA (E. Wake, J.R. Zucker)

**Keywords:** pandemic influenza vaccine, immunization information systems, vaccine reporting, viruses, New York

## Abstract

In 2009, the New York City Department of Health and Mental Hygiene delivered influenza A(H1N1)pdm09 (pH1N1) vaccine to health care providers, who were required to report all administered doses to the Citywide Immunization Registry. Using data from this registry and a provider survey, we estimated the number of all pH1N1 vaccine doses administered. Of 2.8 million doses distributed during October 1, 2009–March 4, 2010, a total of 988,298 doses were administered and reported; another 172,289 doses were administered but not reported, for a total of 1,160,587 doses administered during this period. Reported doses represented an estimated 80%–85% of actual doses administered. Reporting by a wide range of provider types was feasible during a pandemic. Pediatric-care providers had the highest reporting rate (93%). Other private-care providers who routinely did not report vaccinations indicated that they had few, if any, problems, thereby suggesting that mandatory reporting of all vaccines would be feasible.

In April 2009, a novel swine-origin influenza A (H1N1) virus (now called influenza A(H1N1)pdm09 [pH1N1]) was detected in the United States (*1*). During the next 2 months, more than 1 in 10 New York City (NYC) residents reported influenza-like illness; cases occurred primarily among children and young adults (*2*). By June 2009, the World Health Organization had declared an influenza A(H1N1) pandemic (*3*). In July 2009, in anticipation of limited vaccine supply, the Advisory Committee on Immunization Practices and the Centers for Disease Control and Prevention prioritized groups for receipt of monovalent pH1N1 vaccine (*4*,*5*).

Building on previous pandemic influenza preparedness planning, in the summer of 2009, the NYC Department of Health and Mental Hygiene (DOHMH) began planning for pH1N1 vaccine allocation and distribution. To efficiently provide limited doses to a diverse population of providers in a large urban setting, DOHMH developed an allocation plan that included hospitals, private care providers (including adult, pediatric, and obstetric practices), and other outpatient facilities (including federally qualified health centers, pharmacies, DOHMH walk-in immunization clinics, and NYC agencies with a medical unit). In addition, DOHMH conducted a large-scale school-located vaccination program that offered pH1N1 vaccine to virtually all of the 1.4 million NYC schoolchildren in kindergarten through grade 12 (*6*). DOHMH also conducted 58 point-of-dispensing mass vaccination clinics over 5 weekends (*7*).

In NYC, all vaccine doses administered to persons <19 years of age must be reported to the Citywide Immunization Registry (CIR), DOHMH Immunization Information System; this requirement includes influenza vaccine (*8*). Vaccine doses administered to patients >19 years of age can be reported to the CIR with the patient’s consent. Electronic files containing birth certificates are entered into the CIR on a weekly basis to establish a population base and to facilitate reporting among pediatric-care providers. Since 2008, the CIR has been one of the Immunization Information System sentinel sites in the United States and has met data-quality and population-capture requirements, including those of receiving complete and timely data from at least 85% of providers and participation of at least 85% of children <19 years of age (*9*).

On October 28, 2009, because of the pH1N1 pandemic, various provisions of New York State public health law were suspended, including the requirement to obtain consent to report vaccines given to adults. This change authorized the NYC Health Commissioner to issue a Declaration of a Public Health Emergency and to modify the NYC Health Code to require reporting to the CIR of all pH1N1 influenza vaccinations administered, including those administered to persons >19 years of age. This change was made to increase provider accountability, track vaccine uptake, and assist with estimating vaccine coverage.

## Distribution of pH1N1 Vaccine and Reporting of Doses Administered

### Vaccine Ordering and CIR Registration

All medical providers in NYC were invited to order pH1N1 influenza vaccine. Providers who ordered vaccine were required to register with the CIR if they had not previously done so and to electronically sign a memorandum of agreement. Receipt of this memorandum, a faxed vaccine order form, and fulfillment of the order were recorded in the CIR. To rapidly train a large number of providers who served adult patients but had no CIR reporting experience, DOHMH provided instruction by email and through a series of webinars.

Vaccine shipments began in early October 2009. Initial doses were distributed primarily to pediatric-care providers, obstetricians, hospitals (for patients and health care workers), federally qualified health centers, and uniformed personnel (e.g., in fire and police departments). Distribution of doses to adult-care providers and DOHMH walk-in clinics began in the middle of October; most providers received doses at the end of the month. Pharmacies and residential facilities (e.g., nursing homes) began to receive vaccine in mid-December when demand was low. A portion of each weekly shipment was set aside for use in school vaccination programs and weekend point-of-dispensing clinics. Because of the limited supply, providers initially did not receive the full quantity of doses they had ordered; additional vaccine was sent as it became available.

Because vaccine supply was constantly changing and to address the many questions received from the medical community, DOHMH had to maintain continuous 2-way communication with providers throughout the entire period of vaccine allocation and distribution. To that end, the NYC Bureau of Immunization provided weekly email updates to all participating providers and posted these updates on the agency’s public website. DOHMH also held weekly conference calls with private providers, federally qualified health centers, and hospitals to provide updates and answer questions. To ensure that providers were able to contact the Bureau of Immunization with questions or concerns and to check on the status of a vaccine order, a dedicated email account was established so that inquiries could be answered within 48 hours. This email account supplemented the existing Bureau of Immunization hotline, which regularly provides information about vaccines to providers and the public; the hotline was staffed with extra personnel to handle the increased volume of calls.

### Data Collection and Reporting 

Providers could report administration of pH1N1 vaccine doses in 1 of 2 ways: 1) by using the online CIR application, or 2) by sending electronic files, which conformed to a prespecified file format, directly to the CIR. Providers of child and adolescent care were expected to report administration of pH1N1 vaccine doses in the same way that they reported administration of other childhood vaccines. In addition, DOHMH designed a scannable paper reporting form for the school-based vaccination program and point-of-dispensing clinics; the completed forms were scanned, and the data were then automatically uploaded into the CIR.

From October 1, 2009, through March 4, 2010, a total of 988,298 doses of pH1N1 influenza vaccine were reported to the CIR as having been administered. These doses represented 35.5% of the 2,781,700 doses delivered to providers. Reporting to the CIR varied by provider type (Table 1). Those provider types that previously had been required to report to the CIR reported a larger proportion of the pH1N1 vaccine doses received; 53%, 46%, and 43% of total doses received by the Health and Hospitals Corporation (the NYC public hospital system), private pediatric-care providers, and federally qualified health centers, respectively, were reported. Colleges, private hospitals, and adult-care providers reported a smaller percentage of doses received: 29%, 28%, and 20%, respectively. The lowest proportion of doses reported was by residential facilities (4%) and pharmacies (0%). These provider types were the last to receive vaccine in December, when demand was very low citywide.

**Table 1:** Doses of pH1N1 vaccine distributed and reported to the Citywide Immunization Registry, New York, New York, USA,  October 1, 2009–March 4, 2010*

Facility type	No. providers who received pH1N1 vaccine	No. doses received	No. doses administered reported to CIR (% of all vaccine received)
Private care provider			
Adult care	1,471	425,500	84,877 (19.9)
Pediatric care	1,176	635,300	294,324 (46.3)
Private hospital	258	528,300	149,963 (28.4)
Residential facility	129	35,900	3,928 (10.9)
Federally qualified health center	126	132,200	57,345 (43.4)
Health and hospitals corporation†	65	194,300	103,176 (53.1)
Pharmacy	29	139,600	19,679 (14.1)
College	21	18,600	5,459 (29.3)
Uniformed personnel	10	16,000	11,423 (71.4)
DOHMH‡	1	656,000	258,124 (39.3)
Total	3,286	2,781,700	988,298 (35.5)

*pH1N1, influenza A(H1N1)pdm09 virus; CIR, Citywide Immunization Registry; DOHMH, Department of Health and Mental Hygiene.  †New York City public hospital system. ‡Includes all DOHMH-conducted vaccination activities, including walk-in immunization clinics, school vaccination programs, and point-of-dispensing sites.

### Provider Survey

Because a large number of pH1N1 vaccine doses were distributed but not reported to the CIR, the Bureau of Immunization conducted a survey to estimate the number of doses actually administered by providers. The survey, which consisted of 19 questions, sought to identify how many doses providers administered and how many viable doses and expired/spoiled doses were still in stock. Providers were told the number of doses distributed to them and the number reported to the CIR. They were then asked if this was the actual number of doses given and, if not, how many more were given but not reported to the CIR. The survey also included questions about the quality of communications received from DOHMH and about providers’ experiences using the CIR.

All facilities and providers that had ordered pH1N1 vaccine from DOHMH by March 4, 2010, were eligible for inclusion in the survey. DOHMH clinics, point-of-dispensing clinics, and the school vaccination program were excluded because DOHMH conducted these operations. The complete list of potential participants was stratified into 9 groups according to facility type: private adult-care providers, private pediatric-care providers, private hospitals, colleges and universities, federally qualified health centers, the Health and Hospitals Corporation, pharmacies, residential and long-term care facilities, and uniformed personnel. A 12% computerized random sample by facility type resulted in a final pool of 395 potential participants, which included at least 1 provider from each of the 9 facility types.

DOHMH used its in-house call center to conduct the survey from March 15 through April 7, 2010. Call center staff entered responses from providers into the online survey tool Survey Monkey (www.surveymonkey.com). The survey was pre-populated with CIR data about the number of doses ordered and administered by the facility. The Bureau of Immunization trained the call center staff and provided background information on pH1N1 vaccination, DOHMH vaccine distribution methods, and CIR reporting methods. Up to 4 calls were made to each of the selected sites during March and April 2010. After 4 unanswered calls or refusal to participate, facilities were considered nonresponders. The survey took ≈5 minutes to complete.

### Survey Results

Of the 395 facilities sampled, 228 completed the survey (58% response rate) (Table 2). Nearly all providers acknowledged that they did not report a portion of administered doses to the CIR; across all provider groups, 9,297 (8%) of the 116,600 doses received by facilities that responded to the survey were reported as administered but not reported to the CIR. Reporting was achieved across provider types: 79% of private pediatric-care providers, 49% of adult-care providers, and 44% of residential facilities surveyed reported at least some of the administered doses to the CIR.

**Table 2:** Estimates of pH1N1 vaccine administration among surveyed providers, New York, New York, USA, October 1, 2009–March 4, 2010*

Facility type	No. providers selected/no. responders	No. doses rec’d	No. doses rep’d (% of all vaccine rec’d)†	No. doses admin’d but not rep’d	Total no. (%) doses admin’d‡	No. doses remaining§	No. doses not accounted for¶	Max no. doses admin’d#	% All admin’d doses rep’d**
Private provider									
Adult care	176/108	26,800	7,117 (26.6)	5,028	12,145 (45.3)	9,270 (34.6)	5,386	17,531	58.6
Ped. care	141/81	47,000	22,244 (47.3)	1,805	24,049 (51.2)	12,267 (26.1)	10,684	34,733	92.5
Private hospital	32/17	20,800	3,905 (18.8)	1,452	5,357 (25.8)	3,128 (15.0)	12,315	17,672	72.9
Residential facility	15/9	2,700	114 (4.2)	801	915 (33.9)	880 (32.6)	905	1,820	12.5
Federally qualified health center	15/5	2,200	881 (40.0)	11	892 (40.5)	523 (23.8)	785	1,677	98.8
Health and hospitals corporation††	8/4	15,100	4,720 (31.3)	200	4,920 (32.6)	209 (1.4)	9,971	14,891	95.9
Pharmacy	4/2	600	0	0	0	600 (100)	0	0	0
College	3/1	1,000	177 (17.7)	0	177 (17.7)	800 (80.0)	23	200	100
Uniformed personnel	1/1	400	265 (66.3)	0	265 (66.3)	135 (33.8)	0	265	100
Total	395/228	116,600	39,423 (33.8)	9,297	48,720 (41.8)	27,812 (23.9)	40,069	88,789	80.9

*pH1N1, influenza A(H1N1)pdm09 virus; rec’d, received; admin’d, administered; rep’d, reported to Citywide Immunization Registry (CIR); max, maximum; ped., pediatric. †Percentage of all doses reported to CIR = number of doses reported to CIR divided by total number of doses received. ‡Total number of doses administered = sum of doses reported to CIR and number of doses estimated administered but not reported to CIR. §Viable or spoiled/expired. ¶Doses not unaccounted for = number of doses received minus number of doses administered and reported to CIR, number of doses administered but not reported to CIR, and doses remaining and/or expired/spoiled. #Maximum number of doses administered = number of doses received minus number of doses remaining and/or expired/spoiled. **Percentage of all administered doses reported to CIR = number of doses administered and reported to CIR divided by total number of doses administered. ††New York City public hospital system.

For most providers, a large proportion of vaccine remained in storage in their facilities as either viable or expired/spoiled. Among all providers surveyed, nearly 27,812 doses (24% of doses received) remained at the facilities. In addition, 40,069 doses (34% of doses received) were unaccounted for, meaning that they were not reported to the CIR or through the survey as administered and not reported as remaining in storage or expired/spoiled.

If the number of doses the surveyed providers reported that they administered is considered to be a reasonable estimate of the true number of doses administered, then 81% of all doses administered were reported to the CIR. Among private noninstitutional providers, pediatric-care providers reported 93% of all doses administered and adult-care providers reported 59% of all doses administered.

### Estimating Citywide pH1N1 Vaccine Administration

To assess potential discrepancies between the number of pH1N1 vaccine doses reported and the number actually administered by each surveyed provider type, we linked survey data to CIR data. The number of doses administered by survey respondents was calculated by aggregating 1) the total number of doses reported to the CIR for each provider group and 2) the total number of doses reported as administered but not reported to the CIR by that group. The reporting rate (number of doses reported to the CIR divided by the number of doses administered, both those reported to the CIR and those reported as administered but not reported by the provider) was then calculated for each provider group. To estimate the number of doses administered but not reported to the CIR by each provider group, we then applied these rates to the citywide population of providers in each group. These numbers were tallied for an estimate of the total number of doses administered citywide. The analysis was based on vaccine doses administered, not on the number of persons vaccinated. Citywide rates for each provider type were weighted by the number of doses received by each provider type group and then averaged to calculate an overall reporting rate. Frequencies were calculated for responses to questions about the vaccine ordering process and DOHMH communications by facility type.

We estimated that as of March 4, 2010, a total of 1,160,587 doses of pH1N1 vaccine were administered (vs. 988,298 doses reported to CIR) citywide. This number represents 42% of all doses delivered to providers. According to the survey results, an estimated 568,561 doses remained in providers’ offices (viable and/or expired/spoiled) and ≈1 million (36%) of the 2.78 million doses distributed were unaccounted for. If these doses had actually been administered, the number of doses administered could be as high as 2.16 million.

### Provider Satisfaction

Nearly all (96%) providers surveyed found the weekly updates on vaccine ordering and reporting sent by DOHMH to be helpful (Table 3). The percentages of providers reporting problems were 10% for enrolling in the CIR, 3% for completing and submitting the provider agreement, 9% for ordering pH1N1 vaccine, 1% for administering pH1N1 vaccine to patients, and 12% for reporting doses to the CIR (Table 4).The greatest difficulties were encountered by the 9 residential facilities surveyed; 11% reported problems with registration, and 22% reported problems with reporting doses to the CIR (Table 4).

**Table 3:** Provider feedback with regard to Department of Health and Mental Hygiene communications and procedures, New York, New York, USA, October 1, 2009–March 4, 2010

Provider type	No. providers	% Providers						
		Received weekly updates	Found updates helpful	Phoned for more information	Spoke with person	Received helpful information by phone	Sent email	Received helpful information by email
Private								
Adult care	108	73	92	34	92	82	15	88
Pediatric care	81	70	98	41	88	86	30	79
Private hospital	17	53	100	24	100	100	41	71
Residential	9	78	100	11	100	100	33	67
Federally qualified health center	5	20	100	40	100	100	40	50
Health and hospitals corporation†	4	25	100	0	NA	NA	25	100
Pharmacy	2	50	100	50	100	0	0	NA
College	1	100	100	100	100	100	0	NA
Uniformed personnel	1	100	100	0	NA	NA	0	NA
Total	228	69	96	35	91	85	23	79

*NA, not applicable. †New York City public hospital system.

**Table 4:** Provider feedback with regard to problems encountered with regard to pH1N1 vaccine, New York, New York, USA, October 1, 2009–March 4, 2010*

Provider type	No. providers	% Providers who encountered problems				
		CIR registration	Provider agreement	Ordering	Vaccine admin	Reporting
Private						
Adult care	108	13	3	10	1	15
Pediatric care	81	4	1	9	0	11
Private hospital	17	6	0	12	0	0
Residential	9	11	0	0	11	22
Federally qualified health center	5	0	0	0	0	0
Health and hospitals corporation†	4	0	25	0	0	0
Pharmacy	2	50	0	0	0	0
College	1	100	0	0	0	100
Uniformed personnel	1	100	100	0	0	0
Total	228	10	3	9	1	12

*pH1N1, influenza A(H1N1)pdm09 virus; CIR, Citywide Immunization Registry; admin, administration. †New York City public hospital system.

## Implications for Future Pandemic Planning

The results of the survey indicated that a large number of distributed pH1N1 doses were never administered to patients. These results are consistent with other pH1N1 vaccination efforts made in NYC during 2009–2010: only 1 of the 58 weekend point-of-dispensing clinics reached capacity, and uptake at the school-located pH1N1 vaccine program was lower than anticipated. As a result, only half of the doses received by DOHMH were administered and nearly all were reported to the CIR. In June 2010, providers who received pH1N1 vaccines from DOHMH were asked to return unused doses to the Central Vaccine Recovery Program operated by the US Department of Health and Human Services (*10*). The number of doses returned by NYC providers through this program was reported as 537,538 doses (20% of distributed doses), further supporting the conclusion that a large number of received pH1N1 vaccine doses were never administered.

Survey results support the feasibility of requiring providers to report individual-level data about vaccine administration to an Immunization Information System during a pandemic. Although vaccine reporting was a new requirement for adult-care providers, approximately half of adult-care providers and 44% of residential facilities successfully reported to CIR the number of pH1N1 vaccine doses administered. Although these rates may seem lower than those for other provider types, they are an accomplishment, given that nearly all of these providers were first-time CIR users and had only a short time to familiarize themselves with a new electronic system and with the routine practice of reporting vaccine doses administered.

Although the overall rate of reporting to the CIR seemed to be only 35%, survey results indicate that ≈81% of doses administered were reported. Not surprisingly, pediatric-care providers reported nearly all of the vaccine doses they administered (92.5%), and adult-care providers for whom CIR reporting was not previously required reported a smaller percentage of doses administered. We have limited information, but we can understand how providers might have had difficulty reporting if, for example, their practices were overwhelmed with patients who were ill or requesting vaccine. On the contrary, a quick-entry data screen was added to CIR to make reporting easier for providers who had not previously reported. Overall, however, few facilities reported having had difficulty meeting the requirements for obtaining pH1N1 vaccine, including the required reporting of vaccine doses administered. This result is notable, especially given that the new reporting mandate was implemented during an emergency situation.

The variable number of vaccine doses administered by different provider types was associated, in part, with when the facilities received vaccine. Most notably, residential facilities and pharmacies received vaccine late in the season, when vaccine was plentiful and readily available elsewhere and when demand was low.

Without requiring providers to report administration of pH1N1 vaccine doses, the DOHMH is unlikely to have received reports of doses administered to adults. Although CIR reporting was not complete, the vaccine data we received proved valuable in many ways. By having providers enter doses administered in real time, DOHMH was able to replenish providers’ vaccine supplies to keep up with demand and make changes to the vaccine allocation plan on an ongoing basis. In addition, DOHMH was able to better monitor vaccine uptake throughout the pandemic, estimate final vaccine use, population coverage, and vaccine effectiveness (*11*). Had the pandemic been more severe and/or had there been more demand for vaccine, reporting of pH1N1 vaccine doses administered would have been even more useful and would have enabled the public health response to be more effective. The key role played by Immunization Information Systems has been affirmed by the Community Preventive Services Task Force, which identified numerous public health benefits, including increasing coverage, monitoring vaccine uptake and coverage, and evaluating public health responses to outbreaks (*12*).

## Limitations

Interpretation of our survey results has limitations. First, we assumed that survey respondents were similar to nonrespondents and to providers not surveyed. It is possible that they differed; in particular, providers who responded to the survey might have been more likely to have also complied with the reporting requirement. If so, our estimates of doses administered could be high.

Second, the number of doses administered but not reported to the CIR was based on providers’ self-report and was not verifiable. Providers might have intentionally underreported the number of doses they indicated they gave but did not report to the CIR to appear to be in compliance with the reporting requirement. Alternatively, providers might have erroneously underestimated the number of doses administered but not reported because they might have based this estimate on the recent low demand rather than on the higher demand during the fall season. Either way, nearly all providers stated that they had not reported all doses administered despite the reporting requirement.

Third, according to the survey estimates, ≈1 million doses of vaccine were unaccounted for. Some of these doses might have been administered, resulting in an underestimate of the total number of doses administered. As mentioned previously, ≈500,000 doses were returned by NYC providers to the US Department of Health and Human Services Central Vaccine Recovery Program. Although it is not clear whether these doses were considered in our survey to be “unaccounted for,” as doses of vaccine reported to us as remaining in the provider’s office at the time of the survey (either viable or expired/spoiled), it supports the assertion that a large number of vaccine doses distributed to providers were never administered.

Last, we used CIR data as of March 4, 2010, to estimate reporting rates, but the survey was conducted through April 7, 2010. Doses administered during this month-long period could have led to an overestimation of doses administered but not reported. However, only 51,111 additional doses were reported to the CIR during this time by all facilities citywide, which was only 5% more doses than the number reported up to the preceding month. If these additional doses represent 81% of doses truly administered, an additional 11,989 doses were given, for a total of 63,100 doses. Adding these to the 1.16 million doses brings the total estimated doses administered to 1.22 million, an increase that does not substantially change our conclusions.

We also conducted a temporal analysis. This analysis did not indicate a difference in reporting delay in November and in February 2010, although many fewer doses were administered in 2010.

## Lessons Learned

Providers who responded to the survey agreed unanimously that regular communications from DOHMH were extremely helpful. Typical communications provided information about vaccine availability, including availability of specific formulations, and which provider types could receive vaccine. Other information included emphasis on the priority groups targeted for vaccination, reporting requirements, and the need for children <10 years of age to receive 2 doses. On the basis of experience, the Bureau of Immunization has continued to send providers regular influenza season updates on vaccine recommendations and availability.

Whereas the CIR previously focused almost exclusively on pediatric-care providers, pH1N1 reporting resulted in communication and data exchange with adult-care providers. During the 2010–11 influenza season, the Bureau of Immunization used these new relationships to communicate with adult-care providers about seasonal influenza vaccine recommendations and supply. According to data matching to existing provider lists, we estimate having reached ≈50% of private adult-care providers, which might not have been possible had the reporting requirements for pH1N1 vaccine not been implemented during the 2009–10 pandemic. Moreover, these providers reported having had few problems registering for pH1N1 vaccine and using the CIR. This finding, along with the fact that about half of newly reporting providers successfully reported to the CIR the vaccine doses administered, suggests that requiring reporting of all vaccines administered, including those given to adults, is feasible.

Under the Health Information Technology for Economic and Clinical Health Act, eligible health care providers can qualify for incentive payments when they demonstrate their capacity to submit immunization data from a certified electronic health record to an immunization registry (“meaningful use” of an electronic health record) (*13*). Mechanisms established during the pH1N1 pandemic to report to the CIR the number of pH1N1 doses administered have provided a foundation for achieving meaningful use among this provider group, which can subsequently help facilitate rapid reporting during future pandemics.

## Conclusions

We found that influenza vaccine reporting was feasible across all provider types, including those not usually required to report to the CIR. We estimate that 1.16 million doses of pH1N1 vaccine were administered to NYC residents during October 1, 2009–March 4, 2010, with a minimum of 998,298 doses and a maximum of 2.16 million doses having been administered. On the basis of this experience, mandating reporting of adult vaccines should be explored because it has been shown to be feasible, even during a pandemic. Additionally, meaningful use requirements have the potential for improving reporting of vaccines and could also prove beneficial during future emergencies.

## References

[1] Novel Swine-Origin Influenza A. (H1N1) Virus Investigation Team; Dawood FS, Jain S, Finelli L, Shaw MW, Lindstrom S, Garten RJ, et al. Emergence of a novel swine-origin influenza A (H1N1) virus in humans. N Engl J Med. 2009;360:2605–15. Epub 2009 May 7. 10.1056/NEJMoa0903810 19423869

[2] Hadler JL, Konty K, McVeigh KH, Fine A, Eisenhower D, Kerker B, Case-fatality rates based on population estimates of influenza-like illness due to novel H1N1 influenza: New York City, May–June 2009. PLoS ONE. 2010;5:e11677. 10.1371/journal.pone.0011677 20657738 PMC2908148

[3] Writing Committee of the WHO Consultation on Clinical Aspects of Pandemic. (H1N1) 2009 Influenza; Bautista E, Chotpitayasunondh T, Gao Z, Harper SA, Shaw M, Uyeki TM, et al. (H1N1) 2009 Influenza. Clinical aspects of pandemic 2009 influenza A (H1N1) virus infection. N Engl J Med. 2010;362:1708–19. 10.1056/NEJMra1000449 20445182

[4] US Department of Health and Human Services, Centers for Disease Control and Prevention, Advisory Committee on Immunization Practices (ACIP). Summary Report, July 29, 2009, Atlanta, Georgia [cited 2013 Nov 8]. http://www.cdc.gov/vaccines/acip/meetings/downloads/min-archive/min-jul09.pdf

[5] Centers for Disease Control and Prevention. Use of influenza A (H1N1) 2009 monovalent vaccine: recommendations of the Advisory Committee on Immunization Practices (ACIP), 2009. MMWR Recomm Rep. 2009;58(RR-10):1–8 http://www.cdc.gov/mmwr/pdf/rr/rr5810.pdf. 19713882

[6] Narciso HE, Pathela P, Morgenthau BM, Kansagra SM, May L, Scaccia A, Description of a large urban school–located pandemic H1N1 vaccination campaign, New York City 2009–2010. J Urban Health. 2012;89:317–28. 10.1007/s11524-011-9640-z 22318374 PMC3324602

[7] Rinchiuso-Hasselmann A, McKay RL, Williams CA, Starr DT, Morgenthau BM, Zucker JR, Protecting the public from H1N1 through points of dispensing (PODs). Biosecur Bioterror. 2011;9:13–21. 10.1089/bsp.2010.0049 21361797

[8] New York City Department of Health and Mental Hygiene. New York City Health Code, section 11.04 [cited 2013 Jul 22]. http://www.nyc.gov/html/doh/downloads/pdf/cir/healthcode2005.pdf

[9] Centers for Disease Control and Prevention. Q&A about IIS sentinel sites: what are sentinel sites? [cited 2013 Nov 7]. http://www.cdc.gov/vaccines/programs/iis/activities/sentinel-sites.html

[10] Zucker J. Central 2009 H1N1 Influenza Vaccine Recovery Program, June 15, 2010 [letter] [cited 2014 Jan 31]. http://www.nyc.gov/html/doh/downloads/pdf/imm/imm-2009-2010-vaccine-recovery-prov-letter.pdf

[11] Hadler JL, Baker T, Papadouka V, France AM, Zimmerman C, Livingston K, Effectiveness of one dose of 2009 influenza A (H1N1) monovalent vaccine at preventing hospitalization with pandemic H1N1 influenza in children 7 months to 9 years of age, New York City. J Infect Dis. 2012;206:49–55. 10.1093/infdis/jis306 22551808

[12] Community Preventive Services Task Force. Increasing appropriate vaccination: Immunization Information Systems [cited 2013 Nov 13]. http://www.thecommunityguide.org/vaccines/imminfosystems.html

[13] Health IT. gov. HealthIT regulations. Meaningful use regulations [cited 2011 Mar 20]. http://healthit.hhs.gov/portal/server.pt?open=512&objID=2996&mode=2

